# Multistate Outbreak of *Listeria monocytogenes* Infections Linked to Fresh, Soft Hispanic-Style Cheese — United States, 2021

**DOI:** 10.15585/mmwr.mm7121a3

**Published:** 2022-05-27

**Authors:** Alexandra Palacios, Mark Otto, Eileen Flaherty, Michelle M. Boyle, Lenka Malec, Kelsey Holloman, Mabel Low, Allison Wellman, Corinne Newhart, Lauren Gollarza, Tracey Weeks, Anthony Muyombwe, Kristen Lozinak, Erin Kafka, Daniel O'Halloran, Teresa Rozza, David Nicholas, Stranjae Ivory, Katherine Kreil, Jasmine Huffman, Laura Gieraltowski, Amanda Conrad

**Affiliations:** ^1^Division of Foodborne, Waterborne, and Environmental Diseases, National Center for Emerging and Zoonotic Infectious Diseases, CDC; ^2^Oak Ridge Institute for Science and Education, Oak Ridge, Tennessee; ^3^Food and Drug Administration, Silver Spring, Maryland; ^4^Connecticut Department of Public Health; ^5^Maryland Department of Health; ^6^New York City Department of Health and Mental Hygiene, New York; ^7^Virginia Department of Health; ^8^Weems Design Studio, Suwanee, Georgia; ^9^New York State Department of Health; ^10^Department of Epidemiology & Biostatistics, School of Public Health, University at Albany, Rensselaer, New York.

Listeriosis is a serious infection usually caused by eating food contaminated with the bacterium *Listeria monocytogenes.* An estimated 1,600 persons become ill with listeriosis each year, among whom approximately 260 die. Persons at higher risk for listeriosis include pregnant persons and their newborns, adults aged ≥65 years, and persons with weakened immune systems. Persons with invasive listeriosis usually report symptoms starting 1–4 weeks after eating food contaminated with *L. monocytogenes;* however, some persons who become infected have reported symptoms starting as late as 70 days after exposure or as early as the same day of exposure (*1*). On January 29, 2021, PulseNet, the national molecular subtyping surveillance network coordinated by CDC, identified a multistate cluster of three *L. monocytogenes* infections: two from Maryland and one from Connecticut (*2*). CDC, the Food and Drug Administration (FDA), and state and local partners began an investigation on February 1, 2021. A total of 13 outbreak-related cases were eventually identified from four states. All patients reported Hispanic ethnicity; 12 patients were hospitalized, and one died. Rapid food testing and record collection by regulatory agencies enabled investigators to identify a brand of queso fresco made with pasteurized milk as the likely source of the outbreak, leading to an initial product recall on February 19, 2021. Fresh, soft Hispanic-style cheeses made with pasteurized milk are a well-documented source of listeriosis outbreaks. These cheeses can be contaminated with *L. monocytogenes* unless stringent hygienic controls are implemented, and the processing environment is monitored for contamination (*3*). U.S. public health agencies should establish or improve communications, including new methods of disseminating information that also effectively reach Hispanic populations, to emphasize the risk from eating fresh, soft Hispanic-style cheeses, even those made with pasteurized milk.

## Investigation and Results

On February 1, 2021, CDC notified state and federal partners of three listeriosis illnesses from Maryland (two cases) and Connecticut (one case) uploaded to PulseNet within the previous 120 days that were highly related (i.e., within four alleles by whole genome sequencing [WGS]). Specimen collection dates ranged from October 20, 2020, to January 6, 2021. All three patients were hospitalized; no deaths were reported. Patients were aged 45–69 years, and one patient was female. All three patients reported Hispanic ethnicity. State partners interviewed patients or their surrogates using the *Listeria* Initiative questionnaire for hypothesis generation (*4*). All three patients reported consuming fresh, soft Hispanic-style cheeses before becoming ill; two reported consuming queso fresco, a type of fresh, soft Hispanic-style cheese. In this outbreak, a case was defined as an infection in a person with a clinical isolate related within five allele differences by WGS and a specimen collection date from October 20, 2020, to March 17, 2021 (Figure).

**FIGURE:**
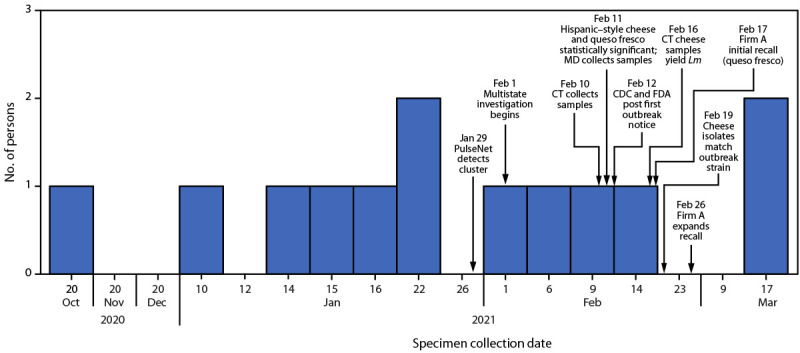
Number of persons infected with the outbreak strain of *Listeria monocytogenes*, by date of specimen collection (n = 13) *—* United States, October 20, 2020–March 17, 2021 **Abbreviations:** CT = Connecticut; FDA = Food and Drug Administration; *Lm* = *Listeria monocytogenes*; MD = Maryland.

Based on food histories from the three index patients, their reported Hispanic ethnicity, and the known association between *L. monocytogenes* and fresh, soft Hispanic-style cheeses, CDC asked the Connecticut Department of Public Health (CDPH) to contact the Connecticut patient for brand information. During re-interview, the patient reported consuming brand A queso fresco. CDC conducted a case-case analysis comparing food exposures for four listeriosis patients included in the outbreak (outbreak cases) with completed *Listeria* Initiative questionnaires to exposures for listeriosis patients not associated with an outbreak by WGS from the same states as outbreak cases (control cases). An exact odds ratio analysis was conducted using SAS software (version 9.4; SAS Institute). Consumption of fresh, soft Hispanic-style cheeses (odds ratio [OR] = 30.4; p<0.001) and queso fresco (OR = 51.2; p = 0.002) were both statistically significant. This activity was reviewed by CDC and was conducted consistent with applicable federal law and CDC policy.*

A total of 13 *L. monocytogenes* infections that met the case definition were reported from four states (Connecticut [one], Maryland [five], New York [four], and Virginia [three]). Patients ranged in age from <1 year to 75 years (median = 51 years). All patients reported Hispanic ethnicity; seven were female. Twelve patients were hospitalized; one died. Four patients became ill during pregnancy, resulting in two pregnancy losses and one premature birth; one patient remained pregnant after becoming ill. Among the eleven patients who completed the *Listeria* Initiative questionnaire, eight reported consuming fresh, soft Hispanic-style cheeses; seven reported consuming queso fresco. Four patients reported consuming brands of cheeses manufactured by firm A, the firm that produces brand A queso fresco.

The Connecticut Food Protection Program and Maryland Rapid Response Team collected samples of fresh, soft Hispanic-style cheeses at stores reported by patients, including brand A queso fresco. Connecticut and Maryland collected and tested 61 fresh, soft Hispanic-style cheese samples; two yielded *L. monocytogenes*. CDPH identified the outbreak strain of *L. monocytogenes* in two samples of brand A queso fresco. WGS analysis of isolates from the CDPH samples showed they were closely related to the clinical isolates (0–4 allele differences), suggesting that patients became ill from brand A queso fresco. All 13 clinical isolates and two cheese isolates were related within five allele differences by WGS.

## Public Health Response

FDA determined that brand A queso fresco was produced by firm A, located in New Jersey, and initiated an inspection. Firm A produced or handled various types of fresh, soft Hispanic-style cheeses under its own brand name and for private label brands. Firm A agreed to recall brand A queso fresco products with expiration dates from February 26 to March 13, 2021. The initial recall and outbreak investigation were announced on February 19, 2021. Because of cross-contamination concerns, firm A agreed on February 26 to expand the recall to all types of Hispanic-style cheeses produced or handled in the facility: queso fresco, requesón, and quesillo. As a result of this investigation, firm A ceased production, repackaging, and distribution of all products manufactured at the facility.

CDC published seven outbreak notices; FDA posted nine outbreak advisories, two recall notices, and two lists of retail establishments that received recalled product. CDC and FDA communications were available in both English and Spanish. In addition, Connecticut published four public communications. Two patients who became ill after the expanded recall, both with specimen collection dates of March 17, 2021, likely purchased and consumed the queso fresco before the recall given their illness dates and the long incubation period for listeriosis (*5*).

## Discussion

Patients in this outbreak were more likely to consume fresh, soft Hispanic-style cheeses, including queso fresco, compared with patients with sporadic *Listeria* infections reported from the same states. In listeriosis outbreaks, prompt, epidemiologically directed food sampling plays a key role in identifying the source of illness. Without the rapid identification of *L. monocytogenes* in firm A’s queso fresco, firm A would not have been identified as the outbreak source as quickly. The public health actions taken within 19 days of cluster identification, firm A’s voluntary recalls, and outbreak notices likely prevented additional illnesses or deaths.

In early 2020, during an unrelated outbreak of listeriosis, *Listeria grayi* and *Listeria innocua,* typically nonpathogenic to humans, were found in firm A’s processing areas. The presence of *Listeria* species in a processing environment indicates that *L. monocytogenes* could survive in that same environment. FDA issued a warning letter to firm A in 2020 because of violations of Current Good Manufacturing Practice regulations and a lack of hazard analysis and preventive control programs (*6*).

Fresh, soft Hispanic-style cheeses made with pasteurized milk continue to constitute a serious risk for listeriosis because cheeses can become contaminated during the production process (after milk pasteurization). High moisture, low salt content, and low acidity support growth of *L. monocytogenes* in these cheeses during refrigerated storage, thereby increasing the risk for illness (*7*). A study of U.S. listeriosis outbreaks associated with soft cheeses during 1998–2014 found that soft cheeses made with pasteurized milk are implicated in more outbreaks than soft cheeses made with unpasteurized milk, which might be related to higher consumption of cheese made with pasteurized milk or to public health messages advising persons at higher risk for listeriosis not to eat cheeses made with unpasteurized milk. Among 17 outbreaks linked to soft cheeses during 1998– 2014, eleven were linked to Hispanic-style cheeses, three of which included cheeses made with unpasteurized milk. The six outbreaks not linked to Hispanic-style cheeses included sheep’s milk, Middle Eastern-style, Eastern European-style, Italian-style, blue-veined, and soft-ripened cheeses (*8*).

Fresh, soft Hispanic-style cheeses, especially those produced in facilities with unhygienic processing conditions, have frequently led to listeriosis outbreaks during the last two decades (*8*). Rapid food testing by regulatory agencies in response to this outbreak investigation identified the implicated cheese. Public health agencies should establish or improve communications, including new methods for disseminating information to emphasize the risk from eating fresh, soft Hispanic-style cheeses, even those made with pasteurized milk, to persons at higher risk for listeriosis, including pregnant persons and their newborns, adults aged ≥65 years, and persons with weakened immune systems.

**Summary**

**What is already known about this topic?**

Listeriosis outbreaks are frequently associated with consumption of fresh, soft Hispanic-style cheeses.

**What is added by this report?**

In early 2021, a multistate outbreak of listeriosis involving 13 cases in four states occurred, resulting in 12 hospitalizations and one death. The outbreak was linked to Hispanic-style cheese within 19 days of cluster detection. Rapid food testing by regulatory agencies in response to the investigation identified the implicated cheese.

**What are the implications for public health practice?**

To prevent severe health outcomes among persons at increased risk for listeriosis, public health agencies should improve communications, including implementing new methods of dissemination to emphasize the risk from eating fresh, soft Hispanic-style cheeses, even those made with pasteurized milk.

## Footnotes

* 45 C.F.R. part 46.102(l)(2), 21 C.F.R. part 56; 42 U.S.C. Sect. 241(d); 5 U.S.C. Sect. 552a; 44 U.S.C. Sect. 3501 et seq.
