# *Erratum:* Vol. 71, No. 21

**DOI:** 10.15585/mmwr.mm7139a6

**Published:** 2022-09-30

In the report on page 709, the title should have read “Multistate Outbreak of *Listeria monocytogenes* Infections Linked to **Queso Fresco** — United States, 2021,” and in the third paragraph, the fifth sentence should have read, “Consumption of **queso fresco (odds ratio [OR] = 51.2; p = 0.002) and other, similar fresh, soft cheeses (OR = 30.4; p<0.001)** were both statistically significant.” On page 710, the first sentence should have read, “Among the eleven patients who completed the Listeria Initiative questionnaire, **seven reported consuming queso fresco; eight reported consuming other, similar fresh, soft cheeses**.” In addition, on page 710, in the first paragraph under “Public Health Response,” the second sentence should have read, “Firm A produced or handled **queso fresco and two similar fresh, soft cheeses (requesón and quesillo)** under its own brand name and for private label brands.” and the fifth sentence should have read, “Because of cross-contamination concerns, firm A agreed on February 26 to expand the recall to **all types of cheese produced or handled in the facility**.” On page 711, in the figure (Figure), the text above the arrow indicating February 11, 2021, should have read, “**Queso fresco and other similar fresh, soft cheeses** statistically significant; MD collects samples.” In addition, on pages 709–712, in the summary box and throughout the text, the terms “**queso fresco**,” “**soft cheese**,” or “**queso fresco and other similar fresh, soft cheeses**” should have been used in place of the term “Hispanic-style cheese.”

**FIGURE:**
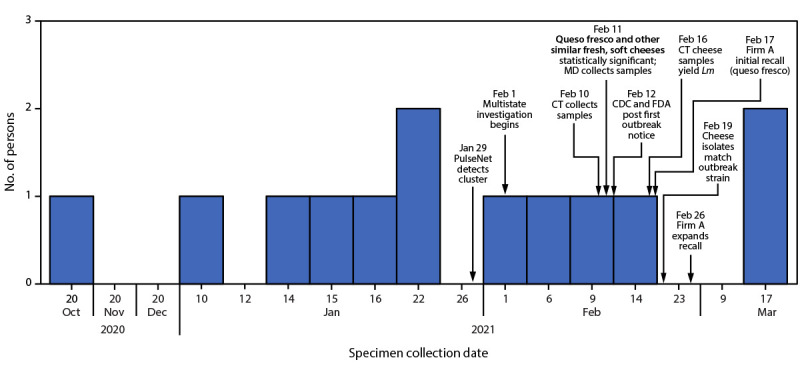
Number of persons infected with the outbreak strain of *Listeria monocytogenes*, by date of specimen collection (n = 13) *—* United States, October 20, 2020–March 17, 2021 **Abbreviations:** CT = Connecticut; FDA = Food and Drug Administration; *Lm* = *Listeria monocytogenes*; MD = Maryland.

